# Automatic classification of ovarian cancer types from cytological images using deep convolutional neural networks

**DOI:** 10.1042/BSR20180289

**Published:** 2018-05-08

**Authors:** Miao Wu, Chuanbo Yan, Huiqiang Liu, Qian Liu

**Affiliations:** 1 College of Medical Engineering and Technology, Xinjiang Medical University, Urumqi 830011, China; 2 Graduate College, Xinjiang Medical University, Urumqi 830011, China

**Keywords:** Classification, Cytological Images, Deep Convolutional Neural Networks, Ovarian Cancer Types

## Abstract

Ovarian cancer is one of the most common gynecologic malignancies. Accurate classification of ovarian cancer types (serous carcinoma, mucous carcinoma, endometrioid carcinoma, transparent cell carcinoma) is an essential part in the different diagnosis. Computer-aided diagnosis (CADx) can provide useful advice for pathologists to determine the diagnosis correctly. In our study, we employed a Deep Convolutional Neural Networks (DCNN) based on AlexNet to automatically classify the different types of ovarian cancers from cytological images. The DCNN consists of five convolutional layers, three max pooling layers, and two full reconnect layers. Then we trained the model by two group input data separately, one was original image data and the other one was augmented image data including image enhancement and image rotation. The testing results are obtained by the method of 10-fold cross-validation, showing that the accuracy of classification models has been improved from 72.76 to 78.20% by using augmented images as training data. The developed scheme was useful for classifying ovarian cancers from cytological images.

## Introduction

Ovarian cancer is the most frequent and aggressive gynecologic cancer [1]. Primary epithelial ovarian carcinoma is subclassified into serous, mucinous, endometrioid, and clear cell subtypes [2]. It is often difficult to precisely differentiate the four subtypes from cytological images only by pathologists’ eyes and mind, especially when a large number of images need to be analyzed and diagnosed, errors can occur. In order to improve the accuracy of diagnosis and reduce pathologists’ workload, we tried to use computer technology in the pathologic diagnosis.

Computer-aided diagnosis (CADx) schemes can potentially make a differential diagnosis more accurate and less dependent on the skill of the observer [3]. With the advent of Whole-Slide Imaging (WSI) and machine learning (ML) algorithms, CADx technology has been greatly developed in recent years. Various studies that apply CADx technology to medical images (such as X-ray, CT, MRI etc.) have been conducted [4–11]. Chang et al. [4] proposed a CADx system to diagnose liver cancer using the features of tumors obtained from multiphase CT images. Nishio and Nagashima [5] developed a CADx system to differentiate between malignant and benign nodules. Yilmaz et al. [6] proposed a decision support system for effective classification of dental periapical cyst and keratocystic odontogenic tumor lesions obtained via cone beam computed tomography. Wang et al. [7] proposed an automatic quantitative image analysis technique of breast cell histopathology images by means of support vector machine (SVM) with chain-like agent genetic algorithm (CAGA). de Carvalho Filho et al. [8] used image processing and pattern recognition techniques to develop a methodology for diagnosis of lung nodules. Alharbi and Tchier [9] designed a CADx system by combining two major methodologies, which are the fuzzy base systems and the evolutionary genetic algorithms. The accuracy of the system can be 97%. Bron et al. [10] used voxel-wise feature maps and SVM to investigate the added diagnostic value of arterial spin labeling and diffusion tensor imaging to structural MRI for computer-aided classification of Alzheimer’s disease, frontotemporal dementia, and controls. Chena et al. [11] established an expert diagnosis system for cerebrovascular diseases and assessed accuracy of the diagnosis system.

From above, we can easily see that ML is widely used in CADx. Amongst them, we found that a branch of ML called deep learning became very popular in medical image processing fields recently. Deep learning is part of a broader family of ML methods based on learning data representations, as opposed to task-specific algorithms. It started from an event in late 2012, when a deep-learning approach based on a convolutional neural network (CNN) won an overwhelming victory in the best-known worldwide computer vision competition [12]. Compared with the traditional medical image processing methods, deep learning such as deep belief nets (DBNs) and deep CNNs uses image pixel values directly as input data instead of image features calculated from segmented objects; thus, manual feature calculation or object segmentation is not required any more, which makes the process more simple and efficient. Since then, researchers in virtually all fields, including medical imaging, have started actively participating in the explosively growing field of deep learning. Xu et al. [13] proposed leveraging Deep CNN (DCNN) activation features to perform classification, segmentation, and visualization in large-scale tissue histopathology images. Teramoto et al. [14] developed an automated classification scheme for lung cancers presented in microscopic images using DCNN. Gao et al. [15] proposed an automatic framework for human epithelial-2 cell image classification by utilizing the DCNNs. The results showed that the system has excellent adaptability and accuracy. Masood et al. [16] proposed a computer-assisted decision support system in pulmonary cancer which was based on deep fully CNN to detect pulmonary nodule into four lung cancer stages. The application of DCNNs to medical images has been increasingly investigated by many groups that have achieved certain degrees of success [17–22].

After consulting a large number of relevant studies, we found that until now no one applied deep learning in ovarian cancer classification. Thus, our study focussed on applying DCNN (one of important deep learning methods for image processing) to automatically classify different ovarian cancer types from a certain number of pathological images. The results of the study are helpful for clinical technologists and pathologists to evaluate malignancies accurately and make correct diagnosis decisions.

## Materials and methods

### Image dataset

Eighty-five (85 specimens in all, 24 serous carcinoma, 22 mucinous carcinoma, 21 endometrioid, and 18 clear cell carcinoma.) qualified Hematoxylin-Eosin (H&E) stained tissue sections of ovarian cancer were obtained from First Affiliated Hospital of Xinjiang Medical University. And the time of making specimens varied from year 2003 to 2016. Each tissue section was clearly marked with the subtype, which was confirmed by at least two pathologists.

**Figure 1:**
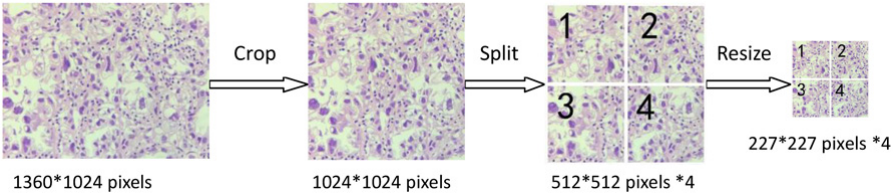
Cytological images preprocessing for automatic classification of ovarian cancer by DCNN

All the H&E stained tissue sections were partly digitized to images in JPG format by a microscope with 40× objective lens (Model: PH100-DB500U-IPL, Brand: Phenix, Place of origin: China) and a digital still camera (Model: Phenix, Brand: MC-D200UVA, Place of origin: China). There were approximately 20–27 qualified images captured from different parts of every H&E tissue section while keeping their orientation invariable. Thus, we finally got 1848 ovarian cancer cytological images, which had uniform matrix size – 1360*1024 pixels. For the requirement of follow-up research, we cropped all the images into 1024*1024 pixels from the center part, each of which was divided into four small images from the center point with the same size of 512*512 pixels, and then resized them to the 227*227 pixels. At last we got 7392 original images with the uniform size of 227*227 pixels. Figure 1 showed the imaging process.

Our study was approved by an established ethics committee and institutional review board. All the tissue sections and other data related to the patients were anonymous.

### Data augmentation

A deep neural network model typically requires a large amount of training data [22].

**Figure 2:**
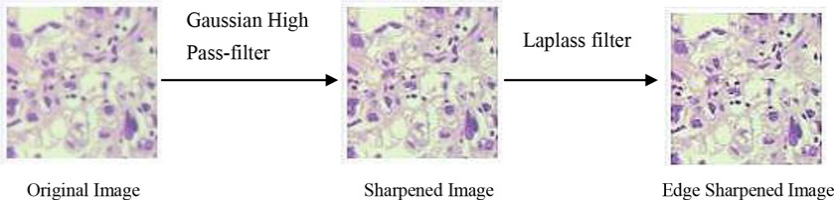
Image enhancement

**Figure 3:**
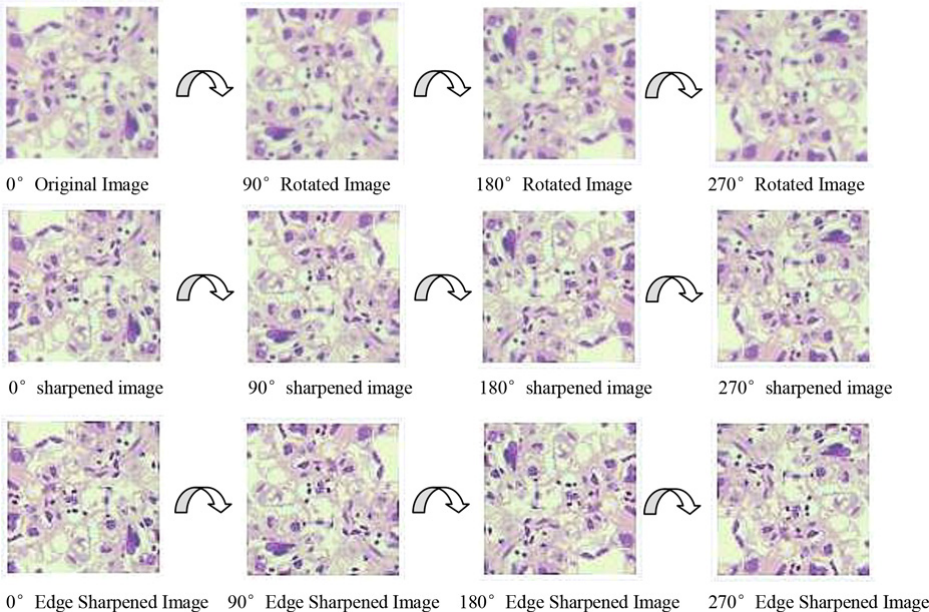
Image rotation

Insufficient size of training sample can directly lead to overfitting and other mistakes. In our study, we increased the sample size by image manipulation in order to improve the accuracy of classification [23,24]. Image manipulation includes image enhancement and image rotation. A Gaussian High Pass-filter with kernel size = 3*3 and Laplass filter were applied to the image to improve the image clarity and edge sharpness. The direction of H&E stained tissue sections was invariable during the image acquisition by the microscope and camera. Thus, we rotated the original images (size: 227*227) from 0° to 270° in 90 steps around their center point to increase the sample sizes. Figures 2 and 3 show the process of image enhancement and rotation.

Two independent recognition models were made by our two group data, one group used original image dataset as training data without image augmentation, and the other one used image dataset augmented as training data, whose sample size was 11 times (81312) bigger than original image sets (7392).

### DCNN architecture

**Figure 4:**
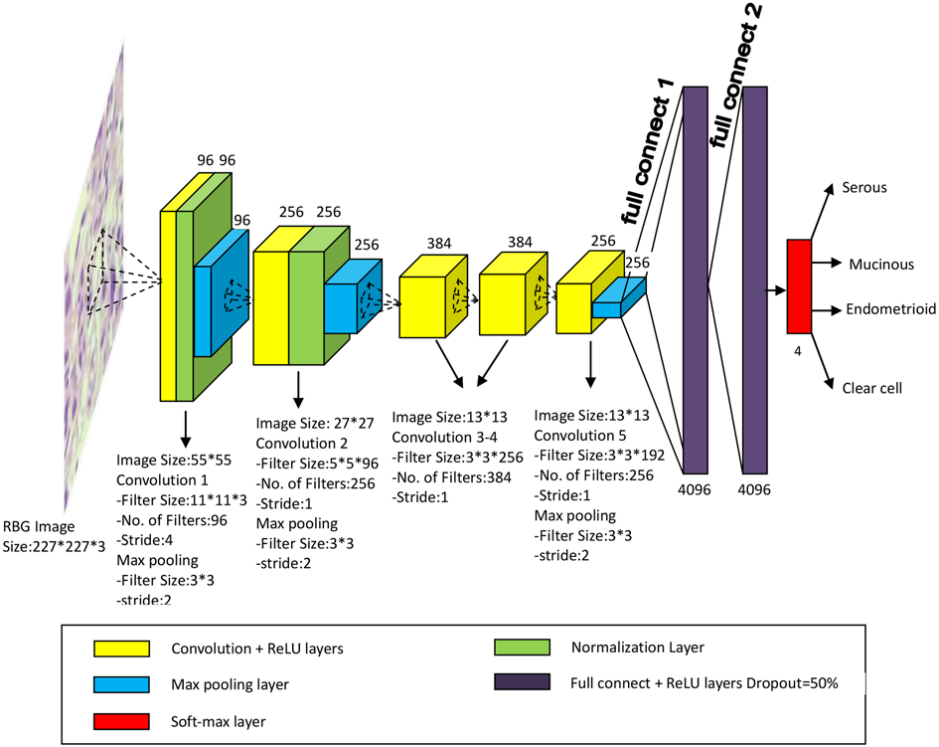
The architecture and illustration of DCNN for ovarian cancer images classification

In our study, we employed a DCNN based on AlexNet to automatic classify the ovarian cancer cytological images. AlexNet, designed by the SuperVision group, consisting of Alex Krizhevsky, Geoffrey Hinton, and Ilya Sutskever, had become well known since it won the first place in the ImageNet Large Scale Visual Recognition Challenge 2012 with a high curacy of image classification [25]. The architecture and illustration of DCNN we built for classifying ovarian cancer types were shown as in Figure 4.

The DCNN for our study had five convolutional layers, three max pooling layers, and two full reconnect layers. Each of the layers was followed by a Rectified Linear Unit (ReLU) as the activation function. Three max pooling layers whose size was 3*3 pixels and stride was 2 were applied to reduce the size of image, which was the input of next convolutional layer. Two full connected layers consisting of a large numbers of the neurones were applied at the end part of the DCNN. Because a fully connected layer occupies most of the parameters, it is prone to overfitting. One method to reduce overfitting was dropout [26-], which was employed in our networks. Dropout is an efficient method for reducing the overfitting, and It is usually used to improve the performance of neural networks on supervised learning tasks in vision, computational biology, document classification, and obtaining state-of-the-art results on many benchmark data sets []. The dropout rate we applied was 50%. The output was the probabilities for four ovarian cancer types, which were calculated by the softmax function.

The DCNN was built by the Caffe package under the Ubuntu 16.04 operation system.

It costed approximately 11 h for training the models by using two graphics cards (Nvidia GeForce GTX 1060, 6 GB memory). The CPU was Intel(R) i5-7500 CUP @ 3.40 GHz and the RAM volume of computer was 4 GB.

## Results

We finally got two independent models of ovarian cancer type classification by training original images (1848 samples) and augmented images (20328 samples) separately. The 10-fold cross-validation was applied to calculate the classification accuracy of the models. The random number of original and augmented images for each dataset is listed in Table 1.

**Table 1:** The number of images in each dataset for 10-fold cross-validation (‘O’ stands for original images and ‘A’ stands for augmented images)

	Serous		Mucinous		Endometrioid		Clear cell	
	O	A	O	A	O	A	O	A
Dataset1	42	462	48	528	42	462	41	451
Dataset2	41	451	50	550	45	495	40	440
Dataset3	54	594	41	451	47	517	40	440
Dataset4	52	572	40	440	54	594	52	572
Dataset5	51	561	44	484	46	506	46	506
Dataset6	52	572	50	550	50	550	42	462
Dataset7	47	517	46	506	47	517	40	440
Dataset8	46	506	41	451	53	583	40	440
Dataset9	48	528	51	561	53	583	45	495
Dataset10	48	528	42	462	47	517	44	484

The classification accuracies of each type in two independent models trained by original images and augmented images were shown in Table 2.

**Table 2:** The classification accuracies for two models

	Original	Augmented
Serous	82.33%	84.14%
Mucinous	71.62%	77.51%
Endometrioid	64.53%	72.93%
Clear cell	72.57%	78.21%
Total	72.76%	78.20%

From Table 2, we can see that the accuracy of classification model trained by augmented image data (78.20%) increased approximately 5.44% compared with the classification model trained by original image data (72.76%). The two models’ architecture are same, however the results are different. It must be caused by the different training data. That indicates image augmentation, including image enhancement and rotation has meaning to the DCNN. Image enhancement, including image sharpening and edge enhancement, make the features of image more prominent. Image rotation amplified the sample size, which directly improved DCNN classification performance.

Table 3 shows our classification model often misclassified endometrioid as serous carcinoma (error rate: 15.11%), mucinous carcinoma as clear cell (error rate: 12.64%), and clear cell carcinoma as mucinous (error rate: 11.39%).

**Table 3:** Confusion matrix of classification results generated by the DCNN model trained and tested by augmented data

	Serous	Mucinous	Endometrioid	Clear cell
Serous	84.14% (4452)	2.34% (124)	6.46% (342)	7.06% (374)
Mucinous	4.21% (210)	77.51% (3862)	5.64% (281)	12.64% (630)
Endometrioid	15.11% (804)	9.70% (516)	72.93% (3883)	2.26% (120)
Clear cell	3.76% (178)	11.39% (539)	6.64% (314)	78.21% (3699)

**Figure 5:**
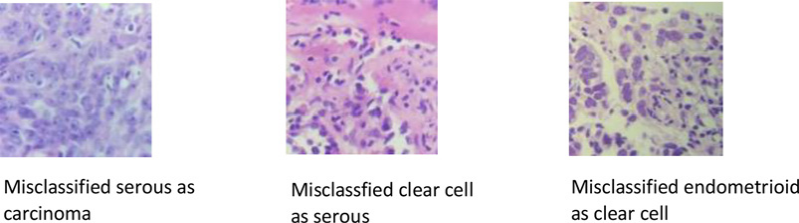
Misclassified ovarian cancer images by DCNN

From Figure 5, we can see most of misclassified images have a common point that the morphological features of the cells are not obvious. Some of them have blurred cell membranes or nuclei. Some of them are overlapped. Some of them are mixed with two types carcinoma cells in one image, which are prone to error.

## Discussion

Different from the traditional methods of image recognition, we built the automatic classification model for four ovarian cancer types from cytological images by DCNN, which directly used the image matrix as input data without processes of object segmentation and feature extraction. Generally speaking, a DCNN with high recognition rate need to be trained by a large number of samples. So we augmented the original images by image enhancement and image rotation, which directly led the amount of samples expended by 11 times. The results showed that the accuracy of our classification model trained by augmented image was 78.20%, which increased by 5.44% compared with the model trained by original images. To validate the statistical significance of two models’ accuracy, we performed paired-sample *t*test using above data. The result showed that two models’ accuracy has a significant difference (*P*<0.05). The increase had statistical significance. The accuracy of the classification model trained by augmented data is close to the pathologist’s diagnosis level, which is considered as a satisfied result. It demonstrated that the DCNN built based on AlexNet can recognize most ovarian cancer cells after training without any prior knowledge of pathology and cryobiology. However, we found some ovarian cancer images are misclassified, most of which have no obvious cell morphology (overlapped cells, poor clarity etc). Further immunohistochemical tests and manual reading by experienced pathologists are needed for those misclassified specimens in order to get the correct diagnosis results. We thought misclassification may be caused by limited number of samples that have unobvious cell morphology. To verify our proposal, we will try to especially increase the sample volume whose cell morphology was poor and train the model again in future study. Not only that, we will try to adjust DCNN architecture (number of convolutional layers and filters, size of max pooling etc.) spired by Teramoto et al. [14] and Miki et al. [27] or apply other networks such as GoogleNet-scratch, VGGS-scratch, etc. to improve the classification accuracy [28] .

## Conclusion

In this preliminary investigation, we applied the DCNN to automatically classify four different ovarian cancer types. By increasing the sample amount by image augmentation, the accuracy of classification models improved from 72.76 to 78.20%. It indicates that the quantity and quality of the images for training DCNN directly affect its classification performance. The classification result can be effectively used as a helpful suggestion for pathologists in clinical diagnosis.

## Abbreviations

CADx: computer-aided diagnosis
CNN: convolutional neural network
DCNN: deep CNN
H&E: Hematoxylin-Eosin
ML: machine learning
SVM: support vector machine
